# Experimental Investigation of the Effect of the Driving Voltage of an Electroadhesion Actuator

**DOI:** 10.3390/ma7074963

**Published:** 2014-06-25

**Authors:** Keng Huat Koh, M. Sreekumar, S. G. Ponnambalam

**Affiliations:** 1 School of Engineering, Monash University Malaysia, Jalan Lagoon Selatan, 47500 Bandar Sunway, Selangor, Malaysia; E-Mail: koh.keng.huat@monash.edu; 2 Indian Institute of Information Technology, Design & Manufacturing (IIITD&M) Kancheepuram, Off Vandalur-Kelambakkam Road, Melakottaiyur, Chennai 600 127, India; E-Mail: msk@iiitdm.ac.in

**Keywords:** electroadhesion actuator, electrostatic force, holding force, leakage current, corona discharge, dielectric actuation

## Abstract

This paper investigates the effect of driving voltage on the attachment force of an electroadhesion actuator, as the existing literature on the saturation of the adhesive force at a higher electric field is incomplete. A new type of electroadhesion actuator using normally available materials, such as aluminum foil, PVC tape and a silicone rubber sheet used for keyboard protection, has been developed with a simple layered structure that is capable of developing adhesive force consistently. The developed actuator is subjected to the experiment for the evaluation of various test surfaces; aluminum, brick, ceramic, concrete and glass. The driving high voltage is varied in steps to determine the characteristics of the output holding force. Results show a quadratic relation between *F* (adhesion force) and *V* (driving voltage) within the 2 kV range. After this range, the *F*-*V* responses consistently show a saturation trend at high electric fields. Next, the concept of the leakage current that can occur in the dielectric material and the corona discharge through air has been introduced. Results show that the voltage level, which corresponds to the beginning of the supply current, matches well with the beginning of the force saturation. With the confirmation of this hypothesis, a working model for electroadhesion actuation is proposed. Based on the experimental results, it is proposed that such a kind of actuator can be driven within a range of optimum high voltage to remain electrically efficient. This practice is recommended for the future design, development and characterization of electroadhesion actuators for robotic applications.

## 1. Introduction

Electroadhesion is a technique available for developing attachment forces, which is similar to popular methods, such as vacuum suction and magnetic adhesion [1]. Electrostatic force is employed in various applications, such as fabric handling [2,3,4], electrostatic chuck (ESC) handling of silicon wafers [5,6], handling rough objects [7,8], levitation and transportation of silicon wafers [9] and electrostatic glass actuators [10]. Apart from this, electroadhesion is used in a complementary manner as reported in the literature, such as assisted wafer bonding [11], tuning the mechanical behavior of structural elements [12] and tuning the frictional damping behavior of structures [13]. It also finds its usage in robotic applications, such as a latching mechanism for modular robots [14,15] and wall climbing robots [16,17,18,19,20,21,22]. Recent efforts have been to optimize the design of the electroadhesion actuator [23,24,25] and to improve its adhesion force with gecko-like dry adhesives [26,27]. The application of electroadhesion is challenging, but the concept of these actuator systems is basically governed by the capacitance model. In order to better understand the model and to explore it further, an electroadhesion actuator using simple materials and a simple structure has been developed. The simplicity of the developed actuator and the experimental results obtained could be used to understand the parameters that govern the electroadhesion force output.

**Figure 1:**
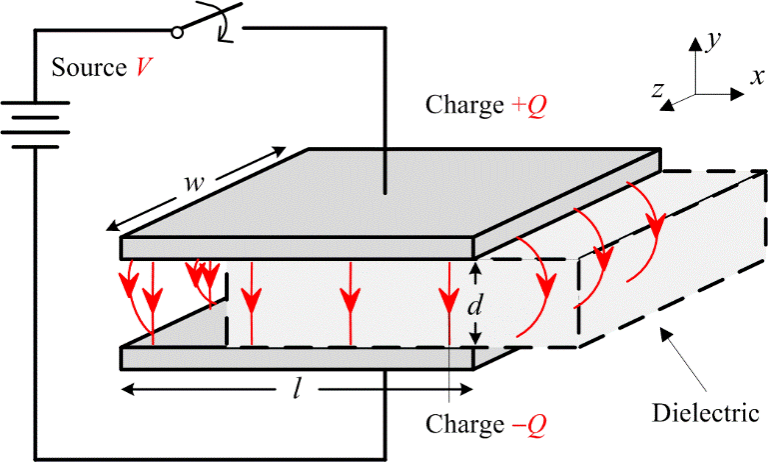
Parallel plate capacitor shown with a linear dielectric partially inserted. Note that the *x-y-z* axes shown here are an essential convention followed throughout this paper. From here, the *x* and *z* directions are considered parallel to the system, while the *y* direction is defined as perpendicular to the system.

The developed actuator model is based on the principle of the parallel plate capacitor shown in Figure 1. Initially, it can be considered as a dielectric removed and left with free space (vacuum) between the plates. The following is a well-known work-energy method used to derive the electrostatic force *F* [28,29]. When the plates are connected to a voltage source *V*, the capacitance developed can be expressed by the standard equation *C =* ε*A/d*, where ε is the permittivity of the material between the electrodes, *A* is the electrode area and d is the distance between the plates. For free space, the permittivity constant ε_0_ (8.85 × 10^−12^ F/m) is assigned to ε. For cases when the free space is replaced by another medium, then ε = ε_0_ε_r_, where ε_r_ is the relative permittivity or dielectric constant of that medium. The energy stored in the capacitor is *W =* 1/2*CV*^2^ or *W =* 1/2*Q*^2^/*C*, depending on the following two situations for the source configuration. 

The first case: if the electrodes are charged and then disconnected from their source (constant charge source), the electric force developed is given as [28,29]:

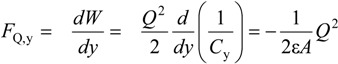
(1)


The negative sign means that the force is attractive, but independent of spacing *d*. If a dielectric is now inserted into the initial free space, then ε is increased and subsequently decreases the attractive force. 

The second case: if the voltage source is switched on and remains connected (constant voltage source), the electric force developed can be expressed as:

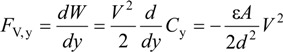
(2)

The negative sign also indicates that the force is attractive and independent of voltage polarity. The force will reach an infinitely large value as *d* approaches zero. If a dielectric is inserted, then ε is increased and subsequently increases the attractive force. 

For both the cases, value of the force developed is the same because of the initial condition *C = Q*_0_/*V*_0_. The second case (constant voltage source) is often adopted as the basis for the development of the electroadhesion actuator model. 

The electric force described in Equation (2) is the component of the vector *F*_V_ in the *y* direction. When a piece of dielectric material is partially inserted between the plates, as shown in Figure 1, polarization will then be in the direction of the electric field *E* and, thus, have *x* and *y* components in the fringing fields near the electrode edges, resulting in a force. The dipoles tend to align themselves. There is a net force towards the –*x* direction, tending to draw the dielectric into the electrodes, because farther ends of the dipoles are in a weaker field. Now, the capacitance can be expressed as [28,29]:

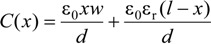
(3)

For a constant voltage source, the electric force is given as:

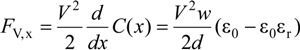
(4)

It can be conveniently expressed as:

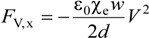
(5)
where χ_e_ is the electric susceptibility of the dielectric, defined as ε_r_ − 1. The force is independent of the voltage polarity and acts in the direction to pull the dielectric into the electrodes. That is, the dielectric will tend to remain within the capacitor plates if an external force is applied in an attempt to pull the dielectric in the +*x* direction. Similarly, pulling the dielectric in the +*z* direction shall result in the capacitor trying to retain it, with the electric force expressed as:

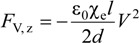
(6)

By comparing Equation (6) with Equation (5), one can see that the resistance to the applied force is direction dependent, where the geometry of the electrode contributes to the magnitude of the force components accordingly. This can also support the results reported in [6], where an interdigitated electrode configuration subjected to drag forces from different shear directions exhibits a different level of resistive force. Furthermore, the magnitude of *F*_V,y_ is much larger than *F*_V,x_ and *F*_V,z_ because of the denominators in Equations (2), (5) and (6).

Once the internal parameters of the actuator (*w*, *l*, *d* and ε_r_) are known, it can be seen from Equations (2), (5) and (6) that *F* is quadratically proportional to the driving voltage *V*. These internal parameters are the material and geometrical properties of the dielectric and electrode. The external parameters are the electric field (driving voltage *V*), the characteristics of the adhered surface (surface roughness, frictional properties, conductivity, *etc*.) and the environmental effects (temperature, humidity, atmospheric pressure, *etc*.). To the best of the authors’ knowledge, the effect of these factors are not yet well understood and modeled. Researchers working on electroadhesion that involves many internal and external parameters are yet to develop a full model incorporating all of the interrelated effects. These parameters and their effects require a series of comprehensive experimental and analytical studies. Fundamental studies are usually conducted on isolated and controlled single parameters to evaluate their individual effects. 

A fundamental understanding of the parameters described above was partly reported in electrostatic wafer chuck (ESC)-related research [5,6]. Here, electrodes were shaped in paired coplanar, comb and spiral patterns and, then, subsequently coated with an insulator layer. It was also noted that there exists a threshold voltage and saturation for the attractive force. However, there was no further discussion or experiments as to why the saturation happened. Similar observations were also found in the structural friction damping study [13], wherein the friction forces obtained (as an evaluation of electrostatic force) were lower than those expected after a certain level of applied electric field. This indicates the saturation nature of the attractive force with the driving voltage. This is due to the effect of the air gap, which requires further investigation. These earlier research works suggested that the *F*-*V* relationship of electroadhesion actuators is not of a pure quadratic nature, as intuitively given by the classic literature. 

The purpose of this study is to further investigate the characteristics of adhesive force in response to a driving voltage. The analysis of other parameters, such as ε, *A* and *d* in Equation (2), as well as the characteristics of the adhered surface and environmental effects are beyond the scope of this paper. The electroadhesion actuator, which is under investigation, is designed with a coplanar electrode pattern and subjected to the assessment of adhesive force under a range of driving voltages. Apart from this introductory section, the other sections of the paper are the construction of the actuator (Section 2), the adhesion mechanism (Section 3), the coupled electromechanical effects (Section 4), the experimental details (Section 5) and the results and discussion (Section 6), followed by the references.

## 2. Construction of the Actuator

**Figure 2:**
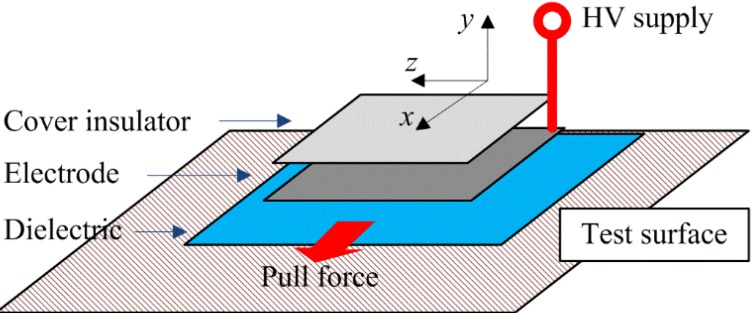
Schematic of the electroadhesion actuator showing an exploded view of the component layers. The direction of the pull force is also shown. HV, high voltage.

The electroadhesion actuator has a generic physical form of the large ratio of its plane area to the thickness. It basically consists of an electrode, dielectric and cover insulator. The components are layered as shown in Figure 2 with the dielectric layer facing the test surface. The controlled parameters are: the thickness of the dielectric, the shape of the electrode and its area. The cover insulator functions as an inhibitor to prevent electrical conductance, which may lead to electrical breakdown during excitation by a HV (high voltage) supply. During testing, an external pull force is applied on this dielectric “backbone” of the actuator. 

There is a wide choice of materials for the components of the developed actuator. Generally, any electrically conductive material is a ready choice for the electrode. Popular choices for a dielectric in electrostatic adhesion research are silicone rubber, Mylar and elastomer [16,26]. Consideration is given for a dielectric material with a high dielectric constant and dielectric strength and the ease of control for fabrication issues. In this work, the materials selected are shown in Table 1 with their respective thickness measured with the thickness gauge 547-400S (Mitutoyo, Kawasaki, Japan). The aluminum foil is Reynolds Wrap^®^, a heavy duty foil made by Reynolds Consumer Products, Lake Forest, IL, USA. The keyboard protector film is VZ-KP1112 made by Vztec, Singapore. The transparency film is Art No. 15110 made by Prosperin Marketing, Rawang, Malaysia. The cover insulator described here is a PVC adhesive tape used to hold the electrode and dielectric in place. All of the described materials are available as ordinary household and office supplies, which is also a key novelty applied to the development of the actuator. They can be substituted by different makes. It is known that the simple construction shown in Figure 2 is basic for electroadhesion actuators. 

**Table 1:** Material combination for an electroadhesion actuator.

Material Combination	A	B
Electrode	Aluminum foil, 24 µm *	Aluminum foil, 24 µm *
Dielectric	Keyboard protector (silicone rubber), 200 µm *	Transparency (cellulose acetate), 90 µm *
Cover insulator	PVC tape, 42 µm *	PVC tape, 42 µm *

* Median values.

**Figure 3:**
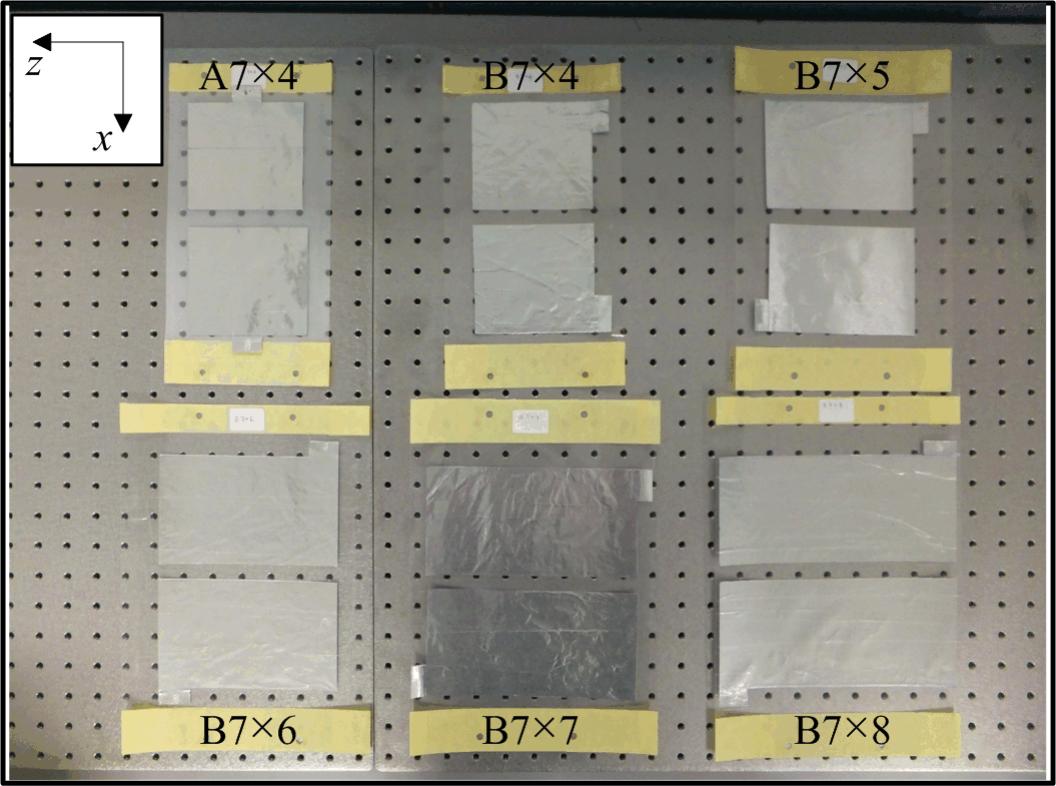
Photograph of the electroadhesion actuators. Naming convention: A7×4, the actuator with material Combination A with an electrode dimension of 7 inches in length and 4 inches in width.

The constructed electroadhesion actuators are shown in Figure 3. The notation “A7×4” means the actuator with material Combination A with an electrode dimension of 7 inches (175 mm) in length and 4 inches (100 mm) in width. A physical gap of 0.5 inches (12 mm) kept the electrodes apart and kept the electrical circuit opened. The difference between material Combinations A and B is only the dielectric material. The simplicity of fabrication enables the good repeatability of the adhesion strength.

## 3. Adhesion Mechanism

**Figure 4:**
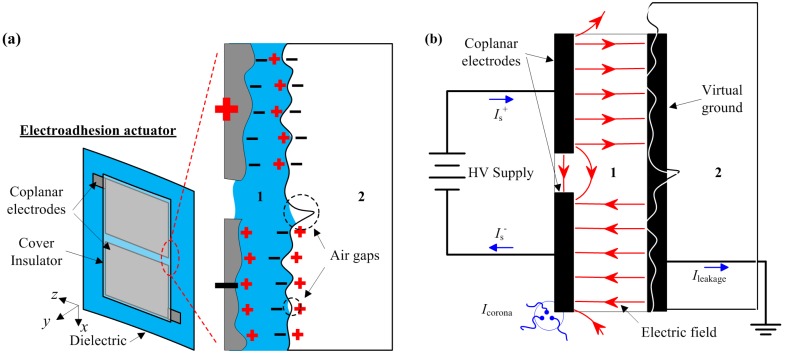
Applying HV to the electroadhesion actuator while placing it on the test surface. (**a**) The induced charge as a result of polarization in dielectric Material 1; (**b**) the electrical schematic and the E field model.

The adhesion mechanism of the fabricated electroadhesion actuator is schematically shown in Figure 4. Here, Medium 1 denotes the dielectric, while Medium 2 denotes the test surface that is positioned on a metal frame touching the floor. The virtual ground plane is assigned to Medium 2, since this electrical node is not an explicit or absolute 0 V potential with reference to the HV (high voltage) supply. By measuring the surface potential of Medium 2 with an electrostatic voltmeter, it is approximately ±200 V in static. This value corresponds to most uncharged objects, which can be treated as the virtual ground in this model. 

In Figure 4a, when the actuator is placed close to the test surface and driven with HV, the electrodes are electrified by conduction, and thus, a free charge presents at the electrodes. Under this electrostatic setup, the electrodes behave similarly to a dipole, where positive and negative charges are separated. Electric charge is then induced at the boundaries of the dielectric layer, which, by means of polarization, resulted in bound charges. 

In Figure 4b, the actuator is modeled as capacitors connected to a voltage source and a virtual ground. Like an RC circuit, the capacitor voltage will reach the HV supply voltage after a time constant. The time constant for the developed electroadhesion actuator is typically less than 1 s. For an ideal electrostatic device, there shall be no further current supplied through the system, *i*.*e*., the supply current *I_s_* become zero. Even though Medium 2 is a conductive test surface, Medium 1 acting as an insulator shall prevent electrical current from conducting, *i*.*e*., block free charges from conducting across Medium 1. According to Kirchhoff’s current law and treating this capacitor system as a super-node:

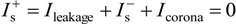
(7)
where *I*_leakage_ is the leakage current out from Medium 2 with the primary path towards the virtual ground; and *I*_corona_ is the discharge current through the air, known as the corona discharge. This discharge happens when the electric field around the electrode is high enough to ionize the atmospheric air, thus forming a conductive region. The location of this discharge usually occurs at the sharp tips and edges of the electrode. 

The dielectric strength of Medium 1 determines the electric field that it can withstand without electrical breakdown. From Equations (2), (5) and (6), it is inferred as *F* ∝ *V*^2^, where *V* charges-up these capacitors. If the HV supply is increased to a high level (not until the breakdown voltage), then one can presume that adhesive force *F* shall follow the quadratic trend. However, at a high electric field, electric conduction in the solid dielectric also takes place, because there is no such thing as a perfect insulator. The theories for high field conduction are given as Schottky, tunneling, Pool–Frenkel, ion or electron hopping and space charge limited [30]. High field conduction is evident in Mylar and Teflon films [31], polyimide films [32,33], polypropylene films [34], polyethylene films [35], *etc*. Over the years, dielectric researchers have extensively summarized the charge transport modeling that occurred in insulating polymers [36]. This charge transport or electrical conduction explains the phenomenon seen as the leakage current described above. It reduces the efficiency of the capacitor system similar to the corona discharge. This reduction of efficiency can be evaluated by measuring the supply current of the electroadhesion actuator driven at a high electric field (*i*.*e*., a higher range of HV supply voltage).

## 4. Coupled Electromechanical Effects

**Figure 5:**
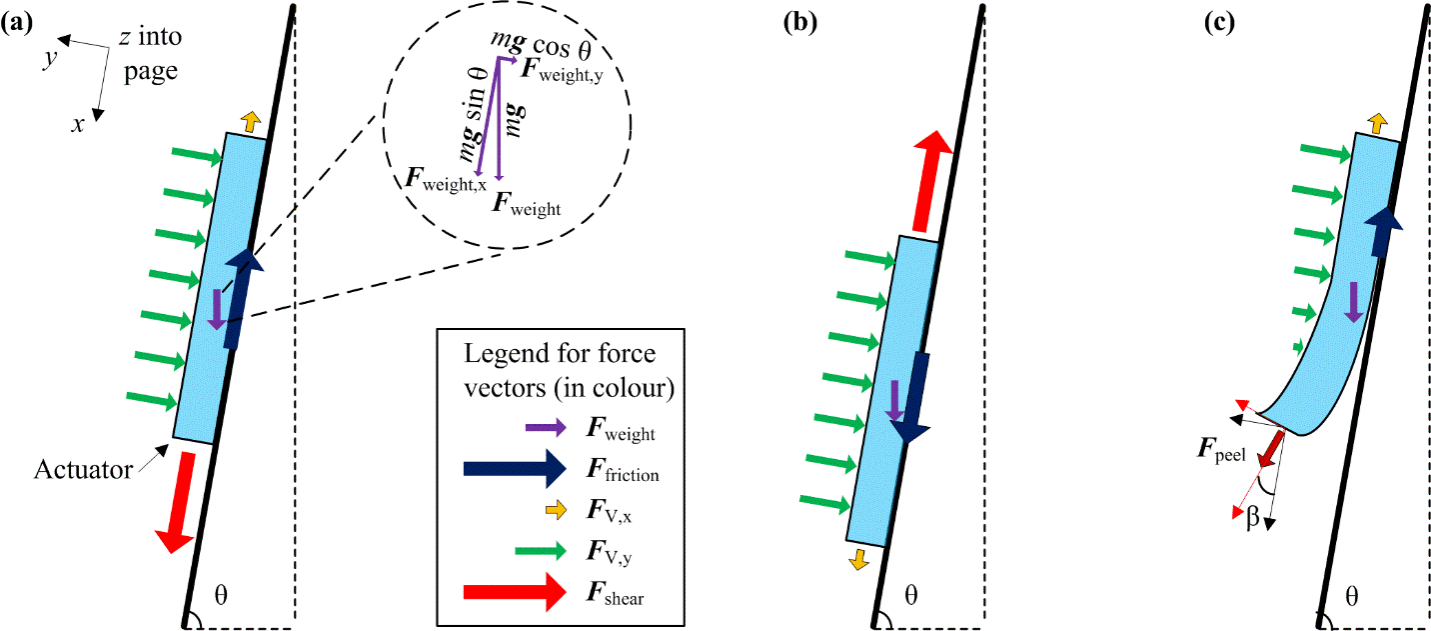
Free body diagrams of the actuator applied to an inclined vertical surface. (**a**) The applied shear force is per the experimental setup (+*x* direction), resulting in *F*_V,x_ and *F*_friction_ opposing this external force; (**b**) if the shear force is applied in the opposite direction, then *F*_V,x_ and *F*_friction_ will result in an opposite direction; (**c**) a rather small peel force at a small angle β will detach the actuator from the surface easily.

The dynamics equivalent of an electroadhesion actuator on the test surface is shown in Figure 5. Free body diagrams for three different applications of external load on the actuator are presented. The parameter *m* is the mass of the actuator, and *g* is the gravitational acceleration. The inclined vertical surface is considered for analysis purpose. Here, *F*_V,y_ is depicted as a uniform force instead of a point force and can be determined using Equation (2). The force vectors are depicted in proportion to their magnitudes, respectively. For example, *F*_weight_ is represented as a shorter vector than *F*_shear_.

In the loading condition, as per Figure 5a, for the actuator to maintain its position (in terms of the *x*-axis):

*F*_shear_ + *F*_weight,x_ ≤ *F*_friction_ + *F*_V,x_ (8)

where *F*_V,x_ is the electrostatic force under constant voltage supply, as in Equation (5). The friction force is an effect of both *F*_V,y_ and the mass effect of the actuator:

*F*_friction_ = µ_s_(*F*_weight,y_ + *F*_V,y_) (9)

where µ_s_ is the coefficient of static friction between the actuator and test surface. Now, Equations (8) and (9) can be expressed as:

*F*_shear_ ≤ µ_s_(*mg* cosθ + *F*_V,y_) + *F*_V,x_ − *mg* sin θ (10)

If θ approaches 0°, then *F*_weight,x_ shall have no effect along the x-axis. If θ approaches 90°, then *F*_friction_ is solely dependent on *F*_V,y_. If *F*_V,y_ and *F*_V,x_ are too weak, then the actuator may slip downwards (+x direction), due to *F*_weight_ alone without the need to apply external load *F*_shear_.

In the loading condition, as per Figure 5b, the direction of *F*_V,x_ is shown in the opposite direction, albeit the same magnitude. One can deduce that *F*_V,x_ is always an opposing force vector to external load. 

In the loading condition, as per Figure 5c, a small magnitude of peel force *F*_peel_ at small angle β can easily detach the actuator from the surface. This is because the magnitude of *F*_V,y_ decreases quickly, due to the rapid increase in the separation distance and also the decrease in the contact area. This is a known failure mode of the electroadhesion mechanism and is analogous to the motion of unzipping a zipper [14]. From this effect, it is deduced that electroadhesion force *F*_V_ works best for holding against external parallel forces (*x*- and *z*-direction), but susceptible to peel forces.

For the experiment, the loading condition, as per Figure 5a, is considered. If *m* of the actuator is low, then both components of *F*_weight_ become negligible compared to external load *F*_shear_. Thus, Equation (10) can be simplified as:

*F*_shear_ ≅ µ_s_*F*_V,y_ + *F*_V,x_ (11)

It can be further approximated to:

*F*_shear_ ≅ µ_s_*F*_V,y_ = *F*_holding_ (12)

since *F*_V,y_ >> *F*_V,x_. The measured external load *F*_shear_ that causes the actuator to shift its position is a close approximation to µ_s_*F*_V,y_. Not to intentionally ignore *m* and *F*_V,x_ this measured holding force (*F*_holding_) is a fair indication of electrostatic force *F*_V_.

## 5. Experimental Setup

**Figure 6:**
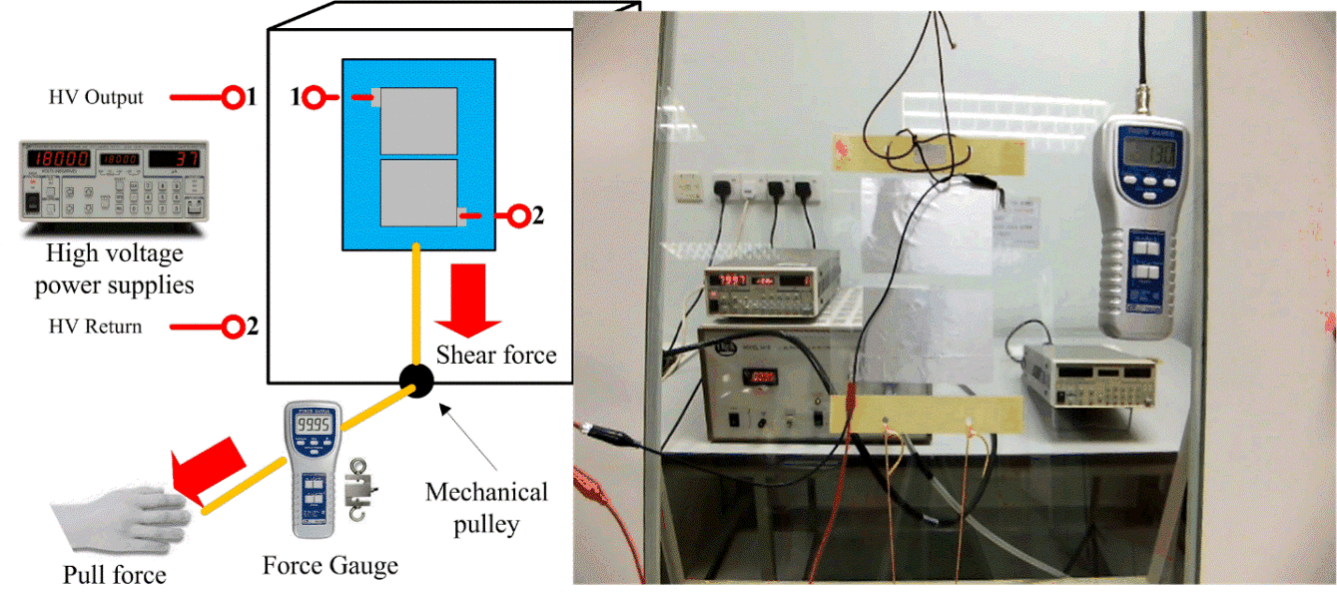
The experimental setup for shear force measurement (a supplemental video for the experiment is available).

Figure 6 shows the experimental setup to measure the important characteristics of the electroadhesion actuator performance, especially the adhesion strength due to *F*_V_ at various supplied voltage *V*.

For the actuator to build-up surface charge, high voltage power supplies PS370 (Stanford Research Systems, Sunnyvale, CA, USA) are connected to the electrodes of the actuator via the HV output and HV return. This instrument is able to supply from 0 V to +20 kV with a 1-V resolution. Supply current can be limited to 0.5 mA, which is equivalent to a maximum of a 10-W power supply. The high voltage power supply equipment is set to automatically trip when the supply current exceeds 100 µA.

As far as the force measurement is concerned, strings are tied to the dielectric backbone of the actuator, which pass through a pulley and then tie onto the force gauge, FG-5100 (Lutron Electronic Enterprise, Taipei, Taiwan). The mechanical pulley functions to restrict the force direction to be parallel to the test surface. The force gauge uses an S-type load cell transducer, which has a resolution of 0.2 N and measurement capacity of 980 N. During the experiment, the peak hold option is chosen to measure the maximum tensile force that the actuator can withstand right before detachment from the test surface. The experiment is conducted using various test surfaces, such as an aluminum plate, a brick surface, ceramic tiles, a concrete slab and a glass panel. The dimension of the test surface board is 1200 mm in height by 900 mm in width. The auxiliary equipment employed is the HV electrostatic voltmeter, Model 341B (Trek, Lockport, NY, USA). This non-contacting surface voltage measurement instrument monitors the surface potentials in the experimental process. The measurement range is 0 to ±20 kV with a 1-V resolution display.

High voltage direct current (HVdc) is applied from 250 V to 20 kV in steps. For each voltage level, tensile force is applied to pull the actuator downwards. Once the actuator moves away from its original position, this means that the *F*_holding_ of the actuator has been overcome by the *F*_shear_ applied. This recorded peak force value of *F*_shear_ is an indication of *F*_holding_, which is an approximation of *F*_V_, as described in Equation (12). After each detachment from the test surface, the actuator requires about 1 min to 5 min to fully neutralize the surface charge accumulated during excitation. This discharge process is longer when HVdc applied is at the higher voltage levels. The discharge was performed by bleeding the remaining charges on the electrode(s) via a resistor to ground, and also physically dragging the actuator in a back-and-forth motion on the test surface. There are 3 trials of measurement for holding force and supply current at each voltage level. The HV electrostatic voltmeter is used to measure the remaining charges on the actuator and test surface and to ensure that the surface charge potential is within ±200 V before the next excitation commences.

Table 2 lists the surface roughness of the test surface and the coefficient of static friction between the actuator and test surface. The surface roughness is evaluated with a portable roughness tester, SJ-210 (Mitutoyo, Kawasaki, Japan), with the measurement settings as follow: *l* = 0.8 mm; *n* = 5; λ_c_ = 2.5 mm, Gauss filter, with stylus speed = 0.5 mm/s. From here, Material A has a high µ_s_ as compared to Material B. Thus, when actuator A7×4 shears horizontally on the aluminum plate, it is expected to have a surface adhesive effect. This is the typical surface characteristic of silicone material. 

**Table 2:** Test surface information (obtained by experiment).

Test surface	Surface roughness Ra *	Coefficient of static friction
Aluminum plate	0.2795	2.36 with Dielectric A
Brick surface	2.6465	0.27 with Dielectric B
Ceramic tiles	0.0295	0.22 with Dielectric B
Concrete slab	6.2935	0.30 with Dielectric B
Glass panel	0.0110	0.22 with Dielectric B

* Median values.

The mass of electroadhesion actuators are listed in Table 3. From these tables, the resultant *F*_weight_ and *F*_friction_ values, as in Equation (10), are negligible when compared to the *F*_holding_ values, which are presented in the Results section (Section 6) next.

**Table 3:** Mass of the electroadhesion actuator.

Actuator	Mass (g)	*mg* as in Equation (10) (N)
A7×4	20.45	0.20
B7×4	10.58	0.10
B7×5	13.70	0.13
B7×6	16.67	0.16
B7×8	18.03	0.18

## 6. Results and Discussion

### 6.1. Characteristics of the F-V Curve and I-V Curve

The electroadhesion actuators responded almost instantly when the HVdc was switched on. They conformed to the test surfaces within a second after the static voltage was generated. A soft high frequency sound was present and audible, and the acoustic level got louder at higher HVdc levels. This indicates that there is a corona discharge through the surrounding air. As the HVdc is further increased, the hissing noise gets louder. 

**Figure 7:**
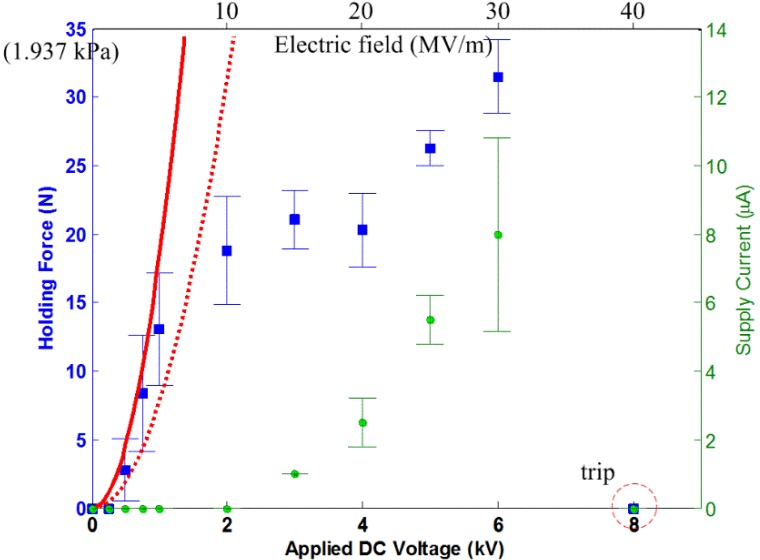
The measured holding force and supply current for the electroadhesion actuator on the aluminum surface, shown with the tripped voltage level. The dashed line describes Equation (2) with the following parameters: ε_r_ = 3.9, *A* = 18.06 × 10^−3^ m^2^, *d* = 200 µm. The solid line describes Equation (12) with µ_s_ = 2.36.

In Figure 7, the experimental data is appended for the actuator, A7×4, on the conductive aluminum plate test surface. It shows that the prediction of *F*_holding_ to within 2 kV is almost achievable. After 2 kV, the quadratic relation in the *F*-*V* curve is no longer valid. The supply current starts to increase quadratically or exponentially. Towards the higher range of voltage levels, *F*_holding_ increases marginally right before the dielectric breakdown at about 8 kV. 

**Figure 8:**
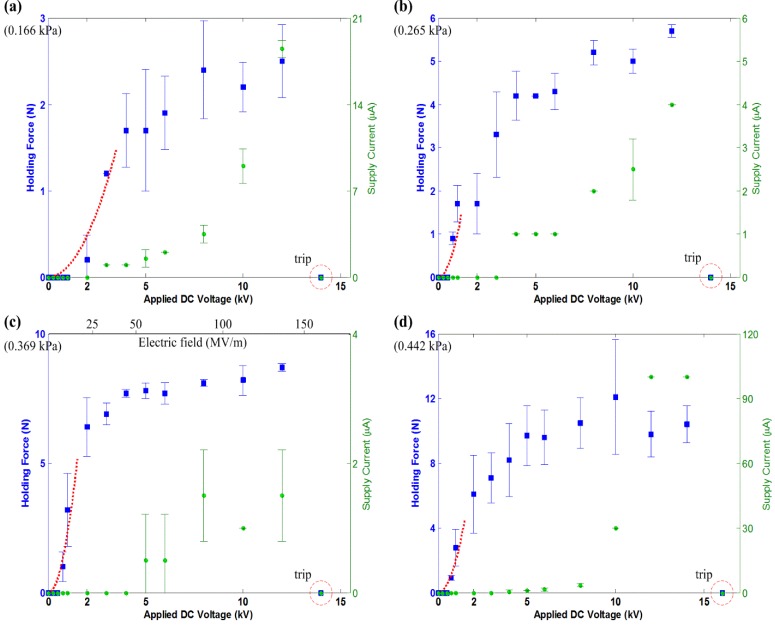
The measured holding force and supply current for electroadhesion actuators on the brick surface, shown with the tripped voltage level. The dashed line describes a quadratic fit over the data, which follows Equation (13). (**a**) B7×4, c = 0.12; (**b**) B7×5, c = 1.0; (**c**) B7×6, c = 2.3; (**d**) B7×8, c = 2.0.

**Figure 9:**
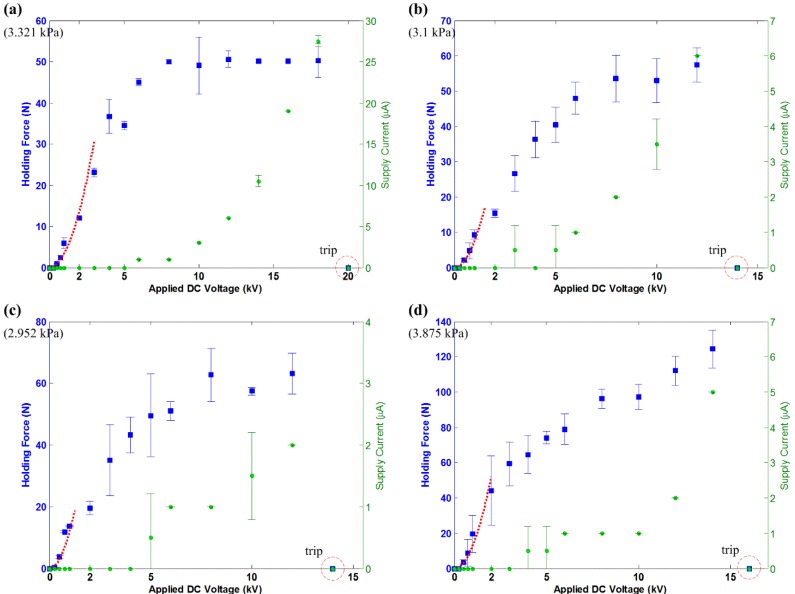
The measured holding force and supply current for the electroadhesion actuators on ceramic tiles, shown with the tripped voltage level. The dashed line describes a quadratic fit over the data, which follows Equation (13). (**a**) B7×4, c = 3.4; (**b**) B7×5, c = 7.5; (**c**) B7×6, c = 12; (**d**) B7×8, c = 13.

**Figure 10:**
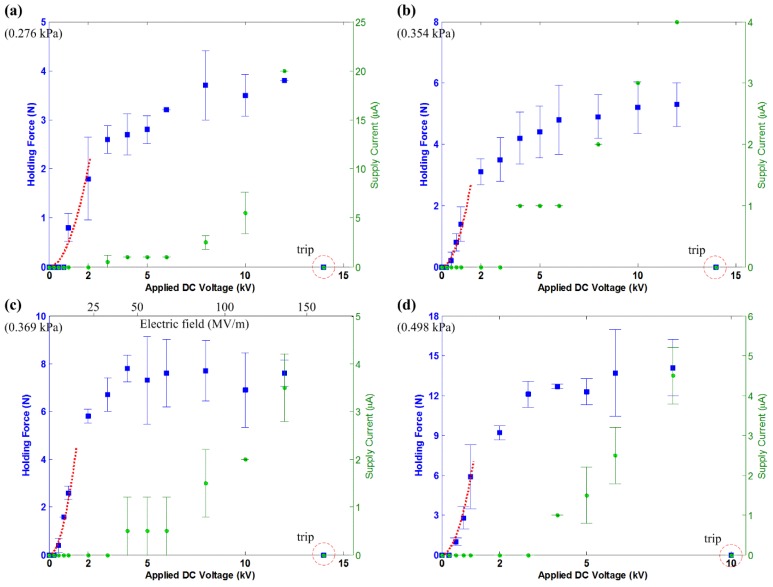
The measured holding force and supply current for the electroadhesion actuators on the concrete slab, shown with the tripped voltage level. The dashed line describes a quadratic fit over the data, which follows Equation (13). (**a**) B7×4, c = 0.5; (**b**) B7×5, c = 1.2; (**c**) B7×6, c = 2.3; (**d**) B7×8, c = 5.8.

**Figure 11:**
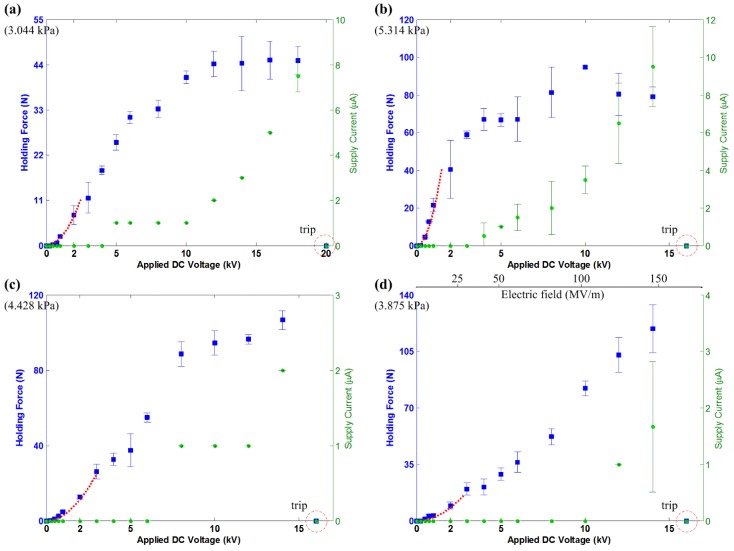
The measured holding force and supply current for the electroadhesion actuators on the glass panel, shown with the tripped voltage level. The dashed line describes a quadratic fit over the data, which follows Equation (13). (**a**) B7×4, c = 1.8; (**b**) B7×5, c = 18; (**c**) B7×6, c = 2.8.; (**d**) B7×8, c = 2.0. (The experiment on glass panel is shown in the supplementary video).

For the actuators, B7×4, B7×5, B7×6 and B7×8, the results are provided for the experiment conducted on the brick surface (Figure 8), ceramic tiles (Figure 9), the concrete slab (Figure 10) and the glass panel (Figure 11). Similarly, these results show a quadratic relation between *F*_holding_ and *V* within the 2-kV range. Note that within this range, there are also more voltage levels for the measured holding force and supply current. A general quadratic fit is provided as:

*F*_holding_ = *k*· *c*· *V^2^* (13)

where *k* = 1 × 10^−6^ (unit-less) and *c* (F/m) is the coefficient of the quadratic relation. The values of *c* for each fit are shown in the figures’ caption for easier reference. Beyond the 2-kV voltage level, *F*_holding_ tends to increase and saturate towards the higher level of the HVdc. In our opinion, this higher range exhibits a square root, cubic root or logarithmic relation between *F*_holding_ and *V*. 

The results also show that a larger electrode area generates higher *F*_holding_. This comes as no surprise if Equations (2) and (5) are referenced. Apart from this, the results show a consistent trend that actuators on ceramic tiles and the glass panel generate much higher *F*_holding_ than on the brick surface and concrete slab. This is due to the surface roughness of the test surfaces (refer Table 2). A smooth surface has more contact area than a rough surface and, thus, increases the electroadhesion strength of the actuator. This is another main characteristic of electroadhesion, and further investigation is necessary to focus on its effects.

The saturation of the *F*-*V* curves is seen in all of the results, which is not an intuition given by Equations (2) and (5). However, then, this outcome is not uncommon, since several reports are readily available to support these results [5,6,13]. The existing model can only predict the quadratic *F*-*V* relation of such actuators within a certain voltage level. After a certain voltage level, the HVdc power supplies start supplying non-zero *I*_s_. According to Equation (7), the ideal electrostatic model is no longer true when *I*_s_ starts to exist. It is shown in the results that *I*-*V* curves start to behave quadratically or exponentially at the voltage level corresponding to where *F*-*V* curves beginning to saturate. The leakage current and corona discharge phenomenon, as explained in Section 3, becomes profound at even a higher electric field.

### 6.2. Proposed Working Model for Electroadhesion Actuator

**Figure 12:**
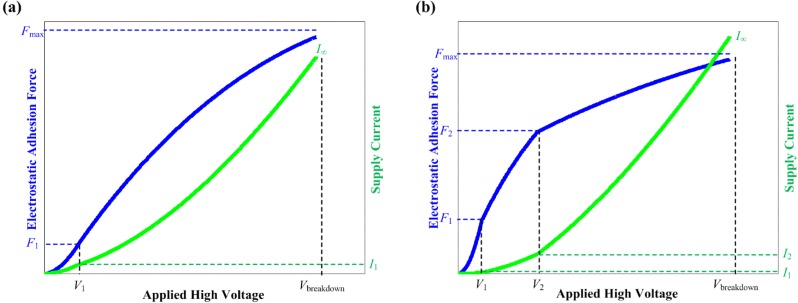
The working model for the electroadhesion actuator depicting (**a**) the second order characteristics and (**b**) the third order characteristics.

Based on the results and discussion, the working models for the electroadhesion mechanism is proposed and shown in Figure 12. These models show the characteristics of electroadhesion actuators in terms of *F*-*V* and *I*-*V* curves. Piecewise functions are appended to describe the behavior at different ranges of voltage levels. For an actuator that exhibits second order characteristics, there are two distinct piecewise functions. Likewise, for an actuator that exhibits third order characteristics, there are three distinct piecewise functions. Indeed, Equation (2) can be regarded as a model for first order characteristic. The modeling accuracy of the electroadhesion actuator can be improved with higher order characteristics. 

Figure 12a shows the second order characteristics model, marked with voltage levels *V*_1_ and *V*_breakdown_, which correspond to *F*_1_ and *F*_max_, accordingly. From 0 V until *V*_1_, the actuator normally exhibits the *F*-*V* curve as predicted by Equation (2). During this stage, the supply current is minimal or almost zero. From *V*_1_ until *V*_breakdown_, the actuator shall exhibit a saturating *F*-*V* curve that has its horizontal asymptote as *F*_max_. During this stage, the supply current increases quickly towards infinity until the point of electrical breakdown. The area under the *I*-*V* curve, *i*.*e*., ∫*I**^.^**dV*, can be calculated to find out the electrical power “consumption” or dissipation of the actuator. 

Figure 12b shares a similar explanation as before. It is recommended to apply HVdc at ≤*V*_2_ to drive the optimum adhesive force out of an electroadhesion actuator. This is because electrifying the actuator at a higher voltage range only increases the adhesive force marginally at the expense of a high supply current. This confirms the hypothesis presented in Section 3 that the saturation of the adhesive force is an indication of the reduced efficiency of the electroadhesion actuator.

## 7. Conclusions

Parametric investigation of an electroadhesion actuator for determining the adhesive force with a driving voltage is presented. The grounds of the evaluation are based on the following factors: the simplicity of the actuator fabrication, the controllable parameters of the experimental method and the repeatability of the experimental results. Firstly, when ordinary conductive and dielectric materials are constructed in a layered structure and actuated by a high voltage, the structure exhibits a surface adhesive effect. Secondly, the results show that this adhesive force *F* obeys the quadratic relation with driving voltage *V*. This good agreement with theoretical predictions is no longer valid at higher fields (beyond 2 kV), where the saturation of the *F-V* responses is observed. Thirdly, it is evident that the force saturation is caused by the leakage current and corona discharge within the dielectric material. Results show that the voltage level that corresponds to the beginning of the supply current matches well with the beginning of the force saturation. Finally, a new working model for electroadhesion is proposed. The actuators shall be driven within a range of optimum high voltage to remain electrically efficient. This practice is useful for future design development and the characterization of electroadhesion actuators.

Future work will require more testing to be performed by proliferating the electrode area, dielectric thickness and also the test surface material. The goal is to develop an empirical model of such an electroadhesion actuator for industrial and robotic applications.

## Supplementary Materials

Supplementary File 1
